# Curing of chronic hepatitis C combined with coronavirus disease 2019 in a couple over 85 years old: a case series study

**DOI:** 10.3389/fmicb.2024.1438827

**Published:** 2024-07-31

**Authors:** Qing-Lei Zeng, Xue-Yan Lv, Ru-Yue Chen, Ya-Jie Pan

**Affiliations:** Department of Infectious Diseases and Hepatology, The First Affiliated Hospital of Zhengzhou University, Zhengzhou, China

**Keywords:** chronic hepatitis C, coronavirus disease 2019, elderly, nirmatrelvir/ritonavir, severe acute respiratory syndrome coronavirus 2, sofosbuvir/velpatasvir

## Abstract

**Introduction:**

Data on the management of patients aged more than 85 years with chronic hepatitis C virus (HCV) and severe acute respiratory syndrome coronavirus 2 (SARS-CoV-2) sequential infections are lacking.

**Methods:**

The current study described the management of an older couple aged more than 85 years with these above-mentioned two diseases treated with 12 weeks of sofosbuvir/velpatasvir (Epclusa^®^) and 5 days of nirmatrelvir/ritonavir (Paxlovid^®^) sequentially. The effectiveness and safety profiles were closely monitored during therapy and till 9 months posttreatment.

**Results:**

In late March 2023, the husband with the main complaint of repeated gingival bleeding and asymptomatic wife were 86 and 85 years old, and had HCV RNA levels of 91,800 and 6,630,000 IU/mL, respectively. On the fourth day of sofosbuvir/velpatasvir treatment, the husband had a moderate headache, and the wife had severe headache and moderate fever and dizziness. We then found that their SARS-CoV-2 test results were positive. After careful consideration, the expert panel decided to treat the couple with oral nirmatrelvir/ritonavir (300 mg/100 mg, twice daily) beginning on the fifth day of sofosbuvir/velpatasvir treatment for 5 days. During the 5 days of nirmatrelvir/ritonavir treatment, the patient’s symptoms and signs gradually improved, and the patient was negative for SARS-CoV-2 RNA on the fifth day of nirmatrelvir/ritonavir therapy. Meanwhile, the husband’s HCV RNA was not detectable after one week of sofosbuvir/velpatasvir treatment till posttreatment month 9, and his ALT level was normal beginning at week 1 of sofosbuvir/velpatasvir treatment. Moreover, the wife’s HCV RNA was not detectable after week 4 of sofosbuvir/velpatasvir treatment till posttreatment month 9. Notably, no other symptoms or signs occurred during the treatment or follow-up period, and other serum biochemical parameters remained stable until 9 months after the discontinuation of sofosbuvir/velpatasvir treatment.

**Conclusion:**

The older couple aged more than 85 years with chronic HCV and SARS-CoV-2 sequential infection were safely cured by the sofosbuvir/velpatasvir and nirmatrelvir/ritonavir sequential treatment.

**Discussion:**

This study suggested that old age should not be a barrier to HCV/SARS-CoV-2 treatment. Given that the proportion of older HCV-infected patients is increasing, clinical trials of direct-acting antiviral agents should include older HCV-infected individuals.

## 1 Introduction

Currently, more than 95% of patients with chronic hepatitis C (CHC) caused by hepatitis C virus (HCV) infection can be cured by using direct-acting antiviral agents (DAAs) (Bhattacharya et al., 2023), except for pregnant women and children younger than 3 years old, who are not recommended for treatment (Zeng et al., 2022). Additionally, elderly CHC patients, especially patients aged more than 85 years, are less common according to the guidelines and related publications (Pawlotsky et al., 2020; Bhattacharya et al., 2023; Pugliese et al., 2023). Moreover, the coronavirus disease 2019 (COVID-19) has affected the world for more than 4 years, and it has become a common disease in real-life settings. To date, no study has evaluated the management strategy of elderly patients who simultaneously or sequentially suffer from CHC and COVID-19.

## 2 Patients and methods

### 2.1 Patients

This study included an elderly couple with CHC. In late March 2023, the husband was admitted to the Hematology Ward due to repeated gingival bleeding for more than 6 months. After admission, his blood platelet count was found to be significantly reduced, and he was diagnosed with CHC based on his previous history of hepatitis C and blood transfusion. A bone marrow biopsy was performed, and no abnormal bone marrow hematopoiesis was found. He was then transferred to the Infectious Disease and Hepatology Unit, where he was diagnosed with CHC-associated compensated cirrhosis. Then, we informed his wife to come for testing, based on his wife’s medical history of hepatitis C, and his wife was also diagnosed with CHC, which was suspected to be infected through sexual transmission.

### 2.2 Comorbidities and management

The husband has a 6-month history of coronary heart disease, for which he was treated with aspirin enteric-coated tablets (100 mg, once daily) starting 4 years ago, but he discontinued this medication 5 months ago due to thrombocytopenia. He has a 2-year history of hypertension, treated with felodipine extended-release tablets (2.5 mg, once daily), and underwent surgery for kidney stones more than 20 years ago. He was recently diagnosed with atrial premature beats. The wife has a 10-year history of coronary heart disease and is regularly taking Tongxinluo (a traditional Chinese medicine) along with aspirin enteric-coated tablets (100 mg, once daily) and atorvastatin calcium tablets (10 mg, once daily). She underwent cataract lens replacement surgery 6 years ago and retinal detachment surgery 4 years ago. Additionally, she was diagnosed with encephalatrophy and lacunar infarction but did not receive any special treatment. In terms of baseline kidney function, the wife had an estimated glomerular filtration rate (eGFR) of 81.79 ml/min/1.73 m^2^, while the husband’s eGFR was 72.676 ml/min/1.73 m^2^. Neither patient had other systemic diseases or liver diseases.

### 2.3 COVID-19 vaccination history

Both the husband and wife were vaccinated with three doses of the Sinovac COVID-19 vaccine (CoronaVac, 0.5 mL per dose, containing 600 SU of inactivated virus antigen) produced by Sinovac Life Sciences Co., Ltd., Beijing, China, in October 2021, November 2021, and April 2022.

### 2.4 Clinical procedures

After careful consideration, the expert panel decided to treat the couple with 12 weeks of sofosbuvir/velpatasvir (Epclusa). The safety and effectiveness of the treatments were closely monitored until at least 3 months after treatment completion. The safety evaluations included any adverse events or fluctuations in the serum test parameters during the treatment and follow-up periods. The effectiveness evaluations included HCV cure (defined as undetectable HCV RNA at 12 weeks after treatment completion) and biochemical response. Serum HCV RNA was monitored using a Roche COBAS^®^ AmpliPrep/COBAS^®^ TaqMan^®^ HCV Test (Roche Molecular Systems; cutoff value, 15 IU/mL) (Zeng et al., 2022). The RT–PCR diagnostic reagents for SARS-CoV-2 infection were obtained from Shanghai BioGerm Medical Biotechnology (China), and suspicious results were confirmed by using a different reagent, i.e., the Guangzhou DAAN GENE Detection Kit for 2019-nCoV (China) (Zeng et al., 2022). A positive result was defined as a cycle threshold of less than 30.

## 3 Results

### 3.1 Baseline characteristics

The husband and wife were 86 and 85 years old, respectively, and had HCV RNA levels of 91,800 and 6,630,000 IU/mL, respectively (Table 1). Moreover, the husband exhibited an elevated alanine aminotransferase (ALT) level of 90 U/L and a reduced albumin level of 32.5 g/L, whereas the wife had normal ALT (25 U/L) and albumin (42.8 g/L) levels. Abdominal ultrasound indicated cirrhosis and splenomegaly in the husband, combined with a liver stiffness measurement (LSM) of 19.3 kPa and a decreased platelet count of 42 × 10^9^/L, so the husband was diagnosed with CHC-associated compensated cirrhosis. Meanwhile, the body mass index of the wife was as high as 31.4, which resulted in LSM testing failure; combined with other parameters, the wife was diagnosed solely with CHC without cirrhosis.

**Table 1:** Baseline characteristics.

Characteristics	Husband	Wife
Gender	Male	Female
Age, years	86	85
Body mass index	22.5 (69/1.75^2^)	31.4 (74.5/1.54^2^)
Main complaint	Gingival bleeding	Abdominal distension
HCV RNA detectable, years	7	4
Suspected transmission route	Transfusion	Sex
HCV RNA, IU/mL	91,800	6,630,000
**Liver function**		
Alanine aminotransferase (0–40 U/L)	90	25
Aspartate aminotransferase (0–40 U/L)	74	24
Alkaline phosphatase (40–130 U/L)	75	29
Gamma-glutamyl transpeptidase (0–58 U/L)	73	82
Total bilirubin (0–17.1 μmol/L)	11.2	11.7
Albumin (35–55 g/L)	32.5	42.8
Alpha fetoprotein (0–9 ng/mL)	6	6.1
PTA (70–150)	74	100
Abdominal ultrasound	Cirrhosis, splenomegaly, fatty liver	Fatty liver
Liver stiffness measurement, kPa	19.3	Cannot be tested due to obesity
Esophageal and gastric varices	No	No
Ascites	No	No
Hepatic encephalopathy	No	No
Chest computerized tomography	Normal	Normal
SARS-CoV-2 RNA	Negative	Negative
**Blood routines**		
White blood cell count (×10^9^/L)	3.5	6.6
Hemoglobin (≥110 g/L)	125	136
Platelets (100–300 × 10^9^/L)	42	223
**Kidney function**		
Creatinine (20–115 μmol/L)	84	57.5
eGFR (ml/min/1.73m^2^)	72.7	81.8
**Lip profiles**		
Total cholesterol (<5.2 mmol/L)	1.71	4.8
Triglyceride (<1.7 mmol/L)	0.67	1.2
High-density lipoprotein (>0.91 mmol/L)	0.80	1.3
Low-density lipoprotein (<3.61 mmol/L)	0.66	3.1
**Glucose**		
Fasting blood glucose (3.6–6.1 mmol/L)	5.8	5.9
Glycosylated hemoglobin (4.0–6.5%)	6.0	5.7
**Myocardial enzyme**		
Creatine kinase (7–200 U/L)	163	24
Creatine kinase-MB (0–25 U/L)	21	13.7
Thyroid function	Normal	Normal
Serum electrolyte levels	Normal	Normal
Bone biopsy	Normal	–
Smoking history, years	50	–
Drinking history, years	50	–
Resolved diseases	Kidney stone	Cataract, amotio retinae
**Comorbidities**		
Hypertension	Yes	No
Coronary heart disease	Yes	Yes
Other disorders	Atrial premature beats	Encephalatrophy, lacunar infarction
Other viral infections	No	No
Other liver diseases	No	No
Treatment regimen for CHC	Sofosbuvir/Velpatasvir (400 mg/100 mg)	Sofosbuvir/Velpatasvir (400 mg/100 mg)
Treatment duration	12 weeks	12 weeks
Treatment regimen for COVID-19	Nirmatrelvir/Ritonavir (300 mg/100 mg)	Nirmatrelvir/Ritonavir (300 mg/100 mg)
Treatment duration	5 days	5 days

CHC, chronic hepatitis C; COVID-19, coronavirus disease 2019; eGFR, estimated glomerular filtration rate; HCV, hepatitis C virus; PTA, Prothrombin time activity percentage; SARS-CoV-2, severe acute respiratory syndrome coronavirus 2.

In early 2023, China ended the “zero COVID-19” policy, but still need to be tested for severe acute respiratory syndrome coronavirus 2 (SARS-CoV-2), especially hospitalized patients who are in high-risk groups. Both of these couples were negative for SARS-CoV-2 at admission, and they did not have a history of COVID-19 during the past 3 years of lockdown due to the “zero COVID-19” policy. The other baseline characteristics of the couples are shown in Table 1.

The couples were placed in a double room ward in the Infectious Disease and Hepatology Unit. In early April 2023, the expert panel decided to treat the couple with oral sofosbuvir/velpatasvir tablets (400 mg/100 mg, once daily, Epclusa^®^) for 12 weeks based on comprehensive judgment of the clinical condition of the couple at the time, the potential long life expectancy, the potential retransmission of HCV between spouses, and the exclusion of drug–drug interactions between hypotensive drugs and sofosbuvir/velpatasvir, including switching the husband’s felodipine extended-release tablets to valsartan tablets (80 mg, once daily) and pausing the wife’s atorvastatin calcium tablets.

### 3.2 Safety and effectiveness

**Figure 1:**
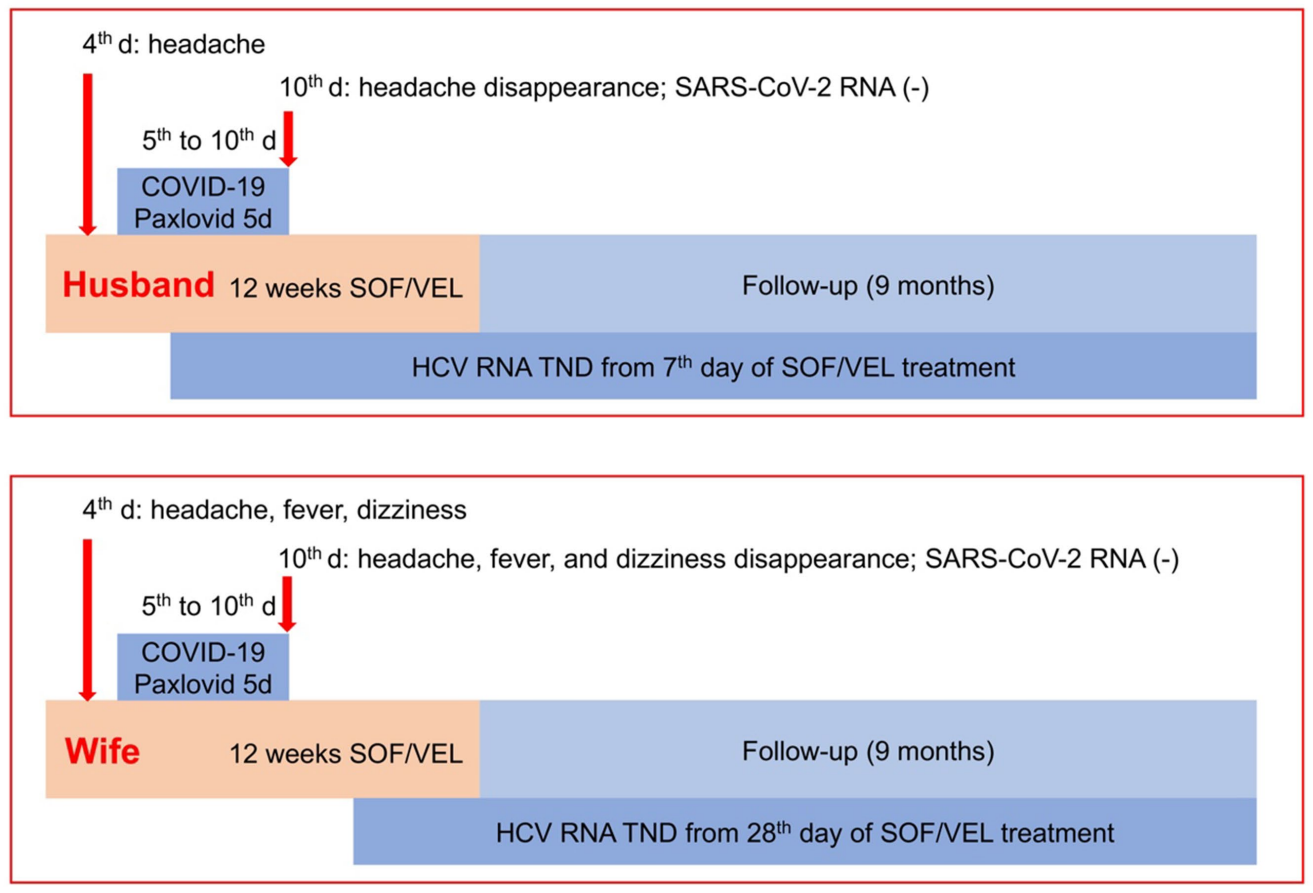
Management strategy and effectiveness. COVID-19, coronavirus disease 2019; HCV, hepatitis C virus; SOF/VEL, sofosbuvir/velpatasvir; TND, target not detected.

On the fourth day of sofosbuvir/velpatasvir treatment, the husband had a moderate headache, and the wife had severe headache and moderate fever and dizziness (Figure 1). First, the expert panel considered that the couple’s headache might be a side effect of sofosbuvir/velpatasvir, but the wife’s fever did not seem to be a side effect of sofosbuvir/velpatasvir. Considering that the “zero COVID-19” policy had just ended at that time and that a large number of people were infected with SARS-CoV-2, we realized that elderly people might be infected with SARS-CoV-2. Therefore, we conducted tests and found that their SARS-CoV-2 test results were positive. For the husband, the cycle threshold values were 26 for the ORF1ab gene and 25 for the N gene; and for the wife, the cycle threshold values were 25 for the ORF1ab gene and 24 for the N gene. Fortunately, chest computerized tomography showed no signs of pneumonia.

There are few reports on how to treat COVID-19 in elderly CHC patients who were receiving sofosbuvir/velpatasvir treatment. Old age is an important risk factor for severe COVID-19, and the husband had a 50-year history of smoking and drinking, although he had quit smoking for 3 years as of his admission to the hospital and had light alcohol consumption previously. After careful consideration, the expert panel decided to treat the couple with oral nirmatrelvir/ritonavir (300 mg/100 mg, twice daily, Paxlovid^®^) beginning on the fifth day of sofosbuvir/velpatasvir treatment for 5 days to decrease the SARS-CoV-2 RNA levels and the risk of severity of COVID-19 (Figure 1).

During the 5 days of nirmatrelvir/ritonavir treatment, the patient’s symptoms and signs gradually improved, and the patient was negative for SARS-CoV-2 RNA on the fifth day of nirmatrelvir/ritonavir therapy (Figure 1), with cycle threshold values of 38 for the ORF1ab gene and 39 for the N gene for the husband, and 37 for the ORF1ab gene and 38 for the N gene for the wife. Additionally, the husband’s HCV RNA was not detectable, and his ALT level was normal beginning at week 1 of sofosbuvir/velpatasvir treatment (Table 2). Moreover, the wife’s HCV RNA was not detectable after week 4 of sofosbuvir/velpatasvir treatment (Table 3). Notably, no other symptoms or signs occurred during the treatment or follow-up period, and other serum biochemical parameters remained stable until week 36, i.e., 24 weeks after the discontinuation of sofosbuvir/velpatasvir treatment (Tables 2, 3). Moreover, the original complaints of the husband and wife, i.e., gingival bleeding and abdominal distension, disappeared at the end of sofosbuvir/velpatasvir treatment and were currently maintained until April 2024.

**Table 2:** Dynamic changes of the husband’s clinical parameters.

Parameters	Week 1	Week 3	Week 4	Week 12	Week 24	Week 48
HCV RNA, IU/mL	TND	TND	TND	TND	TND	TND
Alanine aminotransferase (0–40 U/L)	14	16	15	21	26	45
Aspartate aminotransferase (0–40 U/L)	24	19	20	20	21	39
Alkaline phosphatase (40–130 U/L)	81	81	82	71	74	65
Gamma-glutamyl transpeptidase (0–58 U/L)	64	51	50	33	32	35
Total bilirubin (0–17.1 μmol/L)	12.1	10.4	10.1	8.6	7.1	4.8
Albumin (35–55 g/L)	34.3	33.5	30.9	37.5	37	39.1
Hemoglobin (≥110 g/L)	130	128	130	140	129	134
Platelets (100–300 × 10^9^/L)	52	63	54	72	70	75
Creatinine (20–115 μmol/L)	89	89	88	89	103	103
Estimated glomerular filtration rate (ml/min/1.73m^2^)	67.8	67.7	68.7	67.9	56.4	56.1
Liver stiffness measurement, kPa	–	–	–	–	19.1	–

TND, target not detected.

**Table 3:** Dynamic changes of the wife’s clinical parameters.

Parameters	Week 1	Week 3	Week 4	Week 12	Week 24	Week 48
HCV RNA, IU/mL	27.5	27.4	TND	TND	TND	TND
Alanine aminotransferase (0–40 U/L)	15	23	13	16	13	14
Aspartate aminotransferase (0–40 U/L)	21	21	14	18	19	18
Alkaline phosphatase (40–130 U/L)	73	80	69	90	78	76
Gamma-glutamyl transpeptidase (0–58 U/L)	24	27	21	17	14	27
Total bilirubin (0–17.1 μmol/L)	10.1	11.3	10.1	10.8	9.9	6.6
Albumin (35–55 g/L)	30.9	33.5	30.9	40.5	36	36.6
Hemoglobin (≥110 g/L)	135	133	120	147	127	131
Platelets (100–300 × 10^9^/L)	171	208	173	228	184	182
Creatinine (20–115 μmol/L)	67	68	62	57	57	68
Estimated glomerular filtration rate (ml/min/1.73m^2^)	72.5	71.2	79.1	81.4	81.4	70.2
Liver stiffness measurement, kPa	–	–	–	–	–	–

TND, target not detected.

## 4 Discussion

This study, to the best of our knowledge, is the first to report that elderly individuals aged more than 85 years old with CHC and sequential COVID-19 were cured after being treated with 12 weeks of sofosbuvir/velpatasvir and 5 days of nirmatrelvir/ritonavir. This study indicated that CHC patients should not be excluded based on age, even if they have concomitant COVID-19.

Older age has been associated with an elevated risk of HCV-associated cirrhosis and even hepatocellular carcinoma; however, there are few concerns and published studies on the safety and efficacy of DAAs in older HCV-infected patients (Pugliese et al., 2023). The management of these patients is challenging because of declining kidney function, multiple comorbidities, polypharmacotherapy, and potential drug–drug interactions (Pugliese et al., 2023). Previous study found that the use of sofosbuvir-based DAAs may initially worsen kidney function, but this tends to improve after treatment (Liu et al., 2020). Furthermore, other study has demonstrated that sofosbuvir/velpatasvir is safe and effective for treating hepatitis C patients with stage 4–5 chronic kidney disease (Liu et al., 2022), including those with compensated or decompensated cirrhosis and even patients undergoing dialysis (Huang et al., 2021). In terms of multiple comorbidities and polypharmacotherapy, one study indicated that the HCV cure in patients older than 70 years was lower than that in the general population, and cirrhosis or other comorbidities, such as cardiovascular disease, may be influential factors (Reid et al., 2017; Andres et al., 2019). However, other studies have shown no differences in HCV cure between elderly patients and the general population (Pugliese et al., 2023).

Fortunately, both the husband’s and wife’s kidney function remained stable during the treatment period without significant decline, which is consistent with previous studies (Liu et al., 2020, 2022; Huang et al., 2021). In addition, the CHC husband with the influential factors of cirrhosis and coronary heart disease was cured; notably, his HCV RNA decreased rapidly and was not detected beginning at week 1 of treatment. Moreover, his wife’s HCV RNA concentration seemed difficult to decrease and was undetectable after 4 weeks of treatment. This phenomenon may be caused by the lower baseline HCV RNA level of the husband and higher baseline HCV RNA level of the wife.

Adverse events are also an important concern during CHC treatment in elderly patients. Several studies have also confirmed that side effects during DAAs treatment in elderly patients are mild or no different from those in the general population (Pugliese et al., 2023). Interestingly, there did not seem to be any particular side effects during sofosbuvir/velpatasvir treatment, as headaches, fever, and dizziness may be associated with COVID-19. Therefore, concerns about side effects during DAAs treatment should not be overly focused on for elderly patients.

Potential drug–drug interactions during DAAs treatment should be considered. However, few reports of drug–drug interactions between DAAs and anti-SARS-CoV-2 agents exist. In this study, there seems to be no drug–drug interaction between sofosbuvir/velpatasvir and nirmatrelvir/ritonavir. At the same time, potential drug–drug interactions with nirmatrelvir/ritonavir and sofosbuvir/velpatasvir were successfully avoided by consulting drug interaction websites and discontinuing or replacing medications that posed interaction risks.

Notably, some studies have suggested that sofosbuvir could inhibit SARS-CoV-2’s RNA-dependent RNA polymerase, potentially reducing its activity and thus inhibiting SARS-CoV-2 replication (Izzi et al., 2020; Messina et al., 2022). Other studies indicate that early administration of sofosbuvir/velpatasvir significantly accelerates the clearance of SARS-CoV-2 in patients with mild to moderate COVID-19 and even in those with severe COVID-19 compared to standard care (Sayad et al., 2021; Messina et al., 2022). In our study, the rapid clearance of SARS-CoV-2 suggests that sofosbuvir/velpatasvir may have some inhibitory effect on the virus.

In conclusion, despite the limitations of the small sample size, the management strategy used for the unique CHC couples in this study has important reference significance for the treatment of CHC patients who also have COVID-19. This study suggested that old age should not be a barrier to treatment. Given that the proportion of older HCV-infected patients is increasing, clinical trials of DAAs should include older HCV-infected individuals.

## Data availability statement

The original contributions presented in the study are included in the article/supplementary material, further inquiries can be directed to the corresponding author.

## Ethics statement

The studies involving humans were approved by the ethics committee of The First Affiliated Hospital of Zhengzhou University. The studies were conducted in accordance with the local legislation and institutional requirements. The participants provided their written informed consent to participate in this study.

## Author contributions

Q-LZ: Conceptualization, Data curation, Funding acquisition, Investigation, Methodology, Project administration, Resources, Supervision, Validation, Writing – original draft, Writing – review & editing. X-YL: Investigation, Writing – review & editing. R-YC: Investigation, Writing – review & editing. Y-JP: Investigation, Writing – review & editing.
